# Hahnemann Medical Association of Louisiana

**Published:** 1884-06

**Authors:** 


### HAHNEMANN MEDICAL ASSOCIATION OF LOUISIANA.

At the annual meeting of this association of homœopathic physicians, held April 10th, the following officers were elected: President, S. M. Angell, M. D.; Vice-President, W. Bailey, Jr., M. D.; Recording Secretary F. Engelback, Esq.; Corresponding Secretary, C. J. Lopez, M. D.; Treasurer, Chr. Sanders, M. D.

It was resolved at the same meeting to organize an academy or institute of all homœopathic physicians in the South, the same as exists in the North and West. A meeting for this purpose will be held in this city some time next winter, and a large attendance is expected.

